# Autoregulation and Autoinhibition of the Main NO Synthase Isoforms (Brief Review)

**DOI:** 10.17691/stm2023.15.3.06

**Published:** 2023-05-28

**Authors:** N.A. Popova, S.K. Soodaeva, I.A. Klimanov, V.M. Misharin, A.A. Temnov

**Affiliations:** Researcher, Laboratory of Clinical and Experimental Biophysics; Pulmonology Research Institute, Federal Medical and Biological Agency of Russia, 28 Orekhovy Boulevard, Moscow, 115682, Russia; Researcher, Laboratory of Chemical and Biotechnological Synthesis; Moscow Institute of Physics and Technology (National Research University), 9 Institutskiy Per., Dolgoprudny, Moscow Region, 141701, Russia; Head of Laboratory of Clinical and Experimental Biophysics; Pulmonology Research Institute, Federal Medical and Biological Agency of Russia, 28 Orekhovy Boulevard, Moscow, 115682, Russia; Leading Researcher, Laboratory of Chemical and Biotechnological Synthesis; Moscow Institute of Physics and Technology (National Research University), 9 Institutskiy Per., Dolgoprudny, Moscow Region, 141701, Russia; Senior Researcher, Laboratory of Clinical and Experimental Biophysics; Pulmonology Research Institute, Federal Medical and Biological Agency of Russia, 28 Orekhovy Boulevard, Moscow, 115682, Russia;; Acting Director of the Institute; Pulmonology Research Institute, Federal Medical and Biological Agency of Russia, 28 Orekhovy Boulevard, Moscow, 115682, Russia;; Head of Laboratory of Chemical and Biotechnological Synthesis; Moscow Institute of Physics and Technology (National Research University), 9 Institutskiy Per., Dolgoprudny, Moscow Region, 141701, Russia

**Keywords:** nitric oxide, NO synthase, NOS modeling, NOS autoregulation, autoinhibitory insert, calmodulin, C-tail, electron transfer to NOS

## Abstract

Nitric oxide (II) (NO) is the most important mediator of a wide range of physiological and pathophysiological processes. It is synthesized by NO synthases (NOSs), which have three main isoforms differing from each other in terms of activation and inhibition features, levels of NO production, subcellular localization, etc. At the same time, all isoforms are structurally very similar, and these differences are determined by NOS autoregulatory elements.

The article presents an analysis of the autoregulatory and autoinhibitory mechanisms of the NOS reductase domain that determine differences in the productivity of isoforms, as well as their dependence on the concentration of Ca^2+^ ions. The main regulatory elements in NOS that modulate the electron transfer from flavin to heme include calmodulin (CaM), an autoinhibitory insert (AI), and the C-terminal tail (C-tail). Hydrophobic interactions of CaM with the surface of the NOS oxidase domain are assumed to facilitate electron transfer from flavin mononucleotide (FMN). CaM binding causes a change in the inter-domain distances, a shift of AI and the C-tail, and, as a result, a decrease in their inhibitory effect. CaM also shifts the conformational equilibrium of the reductase domain towards more open conformations, reduces the lifetime of conformations, their stereometric distribution, and accelerates the flow of electrons through the reductase domain. The AI element, apparently, induces a conformational change that hinders electron transfer within the reductase domain, similar to the hinge domain in cytochrome P450. Together with CaM, the C-tail regulates the electron flow between flavins, the distance and relative orientation of isoalloxane rings, and also modulates the electron flow from FMN to the terminal acceptor. Together with the C-tail, AI also predetermines the dependence of neuronal and endothelial forms of NOS on the concentration of Ca^2+^ ions, and the C-tail length affects differences in the productivity of NO synthesis. The inhibitory effect of the C-tail is likely to be reduced by CaM binding due to the C-tail shift due to the electrostatic repulsive forces of the negatively charged phosphate and aspartate residues. The autoregulatory elements of NOS require further study, since the mechanisms of their interaction are complex and multidirectional, and hence provide a wide range of characteristics of the observed isoforms.

## Introduction

Nitric oxide (II) (NO) is the most important intercellular mediator of a wide range of physiological and pathophysiological processes. Thus, it regulates the tone, permeability, and structure of blood vessels, the tone of smooth muscles of internal organs, inflammation and immune response processes, free radical processes, is involved in the pathogenesis of most inflammatory diseases, can serve as a biological marker of inflammation, etc. [1-3].

NO synthases (NOSs), which form NO and are an enzymatic part of a more general NO cycle, are cytochrome-P450-like hemoproteins. They oxidize L-arginine (Arg) in a two-step process including L-hydroxyarginine (NOHA) as an intermediate product. A number of cofactors participate in the catalysis cycle: nicotinamide adenine dinucleotide phosphate (NADPH), flavin adenine dinucleotide (FAD), flavin mononucleotide (FMN), tetrahydropterin, calmodulin (CaM), etc. [4-6].

**Figure 1.**
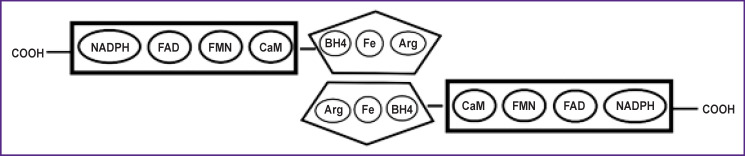
Structural model of NOS [5, 7]

The three main isoforms of NOS (nNOS, eNOS, iNOS) have a number of significant differences: they are encoded by different genes, differ in subcellular localization, NO concentrations produced, and a degree of Ca^2+^ concentration dependence. Nevertheless, the main structural elements of NOS are identical for all isoforms [7-9] (Figure 1).

As it can be seen in Figure 1, the oxygenase domain dimer at the center of NOS contains binding sites for tetrahydrobiopterin (BH4), heme, and L-arginine. The reductase domain, consisting of NADPH, FAD, and FMN, is linked to the oxygenase domain. When bound by an enzyme, CaM is located between FMN and the oxygenase domain [5, 7, 9].

The structural scheme of the three-step model of NOS catalysis that we have proposed before [10] takes into account a significant difference in the rate of reduction of the heme–Fe(II)–O^2^ complex by an electron from the reductase domain and the BH4 coenzyme. The kinetic preference of the second pathway makes the assumption that BH4 works according to binary logic: reduction of the heme–Fe(II)–O^2^ complex, oxidation of the heme–Fe(II) and heme–Fe(II)–NO complexes. The closed cycle of successive oxidation and reduction of BH4 obtained in this case gives a consistent picture of its role in catalysis.

**Figure 2.**
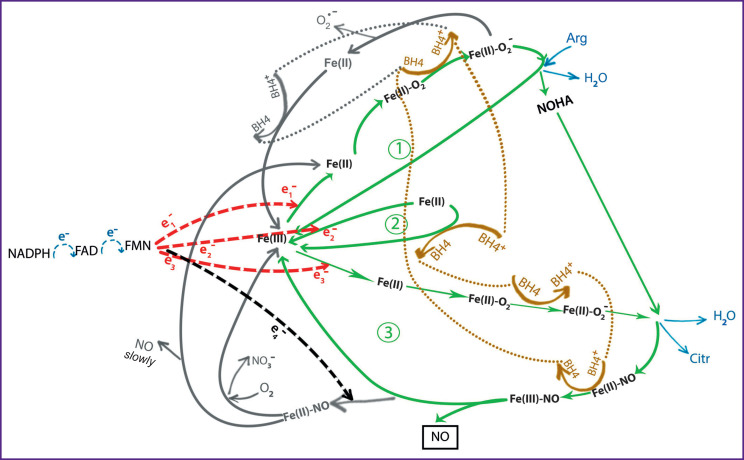
Three-step cycle of NOS catalysis [10]

Figure 2 shows an algorithm for a three-step NOS catalytic cycle under physiological conditions, which was developed for the purpose of subsequent simulation. The normal sequence of the catalytic steps, the result of which is NO synthesizing by the enzyme, is highlighted in green, pathological chains are shown in gray. The closed chain of successive oxidations and reductions of the BH4 coenzyme is highlighted in brown.

The catalytic cycle begins with the transfer of the first electron along the electron transport chain of the reductase domain, then to ferric heme (heme–Fe(III)). According to modern concepts [4, 5], NADPH binds a molecule containing FAD and FMN, reduces FAD, and then the electrons disproportionate between FAD and FMN. CaM binds two Ca^2+^ ions, after which it becomes able to link to the corresponding NOS binding site, but this is not sufficient to initiate conformational changes in the enzyme that allow electron transfer from FMN to the oxygenase domain to ferric heme (hem– Fe(III)). This transfer requires the two remaining CaM binding sites to be also filled with Ca^2+^ ions.

Each step of the three-step cycle of NOS catalysis is triggered by the described process of electron transfer along the electron transport chain of the reductase domain and the reduction of heme–Fe(III) to heme– Fe(II). The steps of a normal NOS productive cycle are shown in Figure 2 in green and under corresponding numbers.

In the first step, heme–Fe(III) is reduced by the first electron to heme–Fe(II), which, if oxygen is sufficient, is oxidized to the heme–Fe(II)–O_2_ complex. In order for this complex to be able to react with the substrate, it is activated by the coenzyme BH4 reduced after the previous _catalytic_ 2 cycle to heme–Fe(II)–O^–^ . In this case, BH4 is converted into the BH4^+^ radical, and the heme– Fe(II)–O^–^_2_ complex is already able to react with arginine, converting it into NOHA with the release of one H_2_O molecule and heme oxidation to its original state (heme– Fe(III)). This ends the first step of the cycle.

The second step is intermediate and serves to restore the BH4 coenzyme oxidized in the previous step. In this case, the second electron from the reductase domain first reduces heme–Fe(III) to heme–Fe(II) similar to the first step, and then the BH4^+^ radical oxidizes heme– Fe(II) to heme–Fe(III), thus reduced for the third step of the catalytic cycle.

In the third step, likewise in the first step, heme–Fe(III) is reduced to heme–Fe(II) by the third electron from the reductase domain, then oxidized to the heme–Fe(II)–O_2_ complex, activated by tetrahydrobiopterin, reduced in the second step, to heme–Fe(II)–O_2_^–^ and reacts with NOHA to form citrulline, an H_2_O molecule, and the heme– Fe(II)–NO complex with an extremely slow release of NO. The BH4^+^ radical formed after the reduction of heme–Fe(II)–O_2_ immediately oxidizes heme–Fe(II)–NO to heme–Fe(III)–NO, which releases NO much faster than its reduced form. In this case, the reduced BH4 becomes ready for the next cycle of catalysis.

If nitric oxide released from the heme–Fe(III)–NO complex has time to leave the enzyme pocket before a new electron arrives, then the catalytic cycle ends productively, and the enzyme becomes ready for a new cycle. Otherwise, the new electron reduces heme– Fe(III)–NO to heme–Fe(II)–NO, which, in case of oxygen sufficiency, turns to NO_3_^–^ and heme–Fe(III), and when oxygen is deficient, it actually blocks the enzyme due to the slow release of NO.

It should be noted that NO catalysis is limited by the slowest step, namely, electron transfer within the reductase domain, as well as from FMN to heme. This transfer is necessary for the reduction of heme–Fe(III) at the beginning of each step of the cycle. Accordingly, the regulation of the rate of this transfer is responsible for the “performance” of NOS in the normal catalytic cycle, and the regulatory mechanisms are extremely important for understanding the functioning of the enzyme. Moreover, in the case of NOS, it is the autoregulatory mechanisms of electron transfer in the reductase domain that determine and explain the difference among different isoforms in the productivity of NO synthesis and in dependence on the Ca^2+^ ion concentration.

**The aim of this review** is to analyze the results of studies of the basic autoregulatory mechanisms of the reductase domain of NOS containing calmodulin (CaM), the autoinhibitory insert (AI), and the C-terminal tail (C-tail). To date, the mechanisms of their functioning have not been sufficiently studied, but from the available data it might be concluded that a complex, multidirectional interaction algorithm provides a wide range of characteristics of the observed isoforms.

## Materials and Methods

The search engines https://scholar.google.ru/ and the PubMed database (https://pubmed.ncbi.nlm.nih.gov/) were chosen as the main source of scientific data. The search depth was 33 years (from 1990 to 2023), the holographic data analysis method was applied. The following words were used as key words: “NOS”, “NO synthase”, “nitric-oxide synthase”, “electron flow”, “electron transfer”, “regulation”, “autoinhibition”, “modulation by”, “conformational control”, “conformational dynamics”, “C-tail”, “C-terminus”, “cytochrome C-reduction”, “ferricyanide reduction”, “autoinhibitory insert”. The total number of sources with the combination of key words [(“NOS” OR “NO synthase” OR “nitric-oxide synthase”) AND (“electron flow” OR “electron transfer”) AND (“regulation” OR “autoinhibition” OR “conformational control” OR “modulation by” OR “conformational dynamics”) AND (“C-tail” OR “C-terminus”)] amounted to 3100 in https://scholar.google.ru/ with very low relevance (only 6 papers cover the issue under study) and only 11 papers in PubMed, only 4 of which are sufficiently relevant. 247 sources with combinations of [(“NOS” OR “NO synthase” OR “nitric-oxide synthase”) AND (“regulation” OR “autoinhibition” OR “modulation by”) AND (“C-tail” OR “C-terminus”) AND (“cytochrome C-reduction” OR “ferricyanide reduction”)] were found in https://scholar.google.ru/ and they had low relevance, and no papers were found in the PubMed database. At the same time, most papers analyze the regulatory role of calmodulin, while other autoregulatory elements are only mentioned. Thus, 3 works have been found, in which the regulatory role of the autoinhibitory insert and C-tail is quantitatively assessed using mutant forms of enzymes. It should be noted that these are works of 1999–2001, and they are not consistent enough on a number of data, and their design fits more pilot studies.

An extremely small number of highly relevant works on this issue indicates that the mechanisms of autoregulation of NO synthases are currently a poorly studied area, the amount of existing experimental quantitative estimates is not sufficient for the adequate understanding of the activity of autoinhibitory and regulatory elements, and for their modeling as well.

## Regulatory functions of calmodulin and Ca^2+^

The calmodulin protein plays a key role in the regulation of electron transfer in NOS. When two of the four CaM binding sites with higher affinity are filled with Ca^2+^, CaM binds to NOS, located, as it is commonly assumed, between the FMN and the heme. This initiates a conformational change that accelerates electron transfer within the reductase domain: from NADPH to FAD, from FAD to FMN. After the two remaining free binding sites are filled with Ca^2+^, CaM accelerates electron transfer from FMN to heme [4].

According to the available data [4], the mechanisms of removal of repression at the key stages of electron transfer are not associated with changes in the thermodynamics of reactions. Since the reductase domain can exist in any state, from one- to four-electron reduced, as a dynamic system of open and closed conformations, the main mechanisms of the regulatory influence of CaM are assumed to be as follows:

shifting conformational equilibrium towards more open conformations;

reduction of the lifetime of conformations and acceleration of transitions between them;

narrowing stereometric distributions of conformations;

restriction of degrees of freedom within the enzyme, which directs the movement of the FMN domain in space.

The above mechanisms are most likely due to the ability of CaM to form ionic bonds and hydrophobic interactions with the NOS oxidase domain, which helps to guide FMN domain docking and forms an optimal electron pathway. CaM presumably also removes the inhibitory effect of AI on the FMN–heme docking region [4, 11, 12].

In the absence of CaM, nNOS practically does not synthesize NO (concentrations are not determined), and eNOS produces NO at a level of 2.5% of the level of synthesis in the presence of CaM. The filling of the CaM binding sites with Ca^2+^ ions actually triggers electron transfer to heme, most likely due to the shift of the inhibitory regulatory elements: AI and the C-tail. At the same time, iNOS, unlike the other two isoforms, does not have AI and is immediately expressed with CaM, which makes it practically independent of the Ca^2+^ ion concentration [11].

## Regulatory functions of the autoinhibitory insert

The main difference in the structure of the three main mammalian NOS isoforms is the presence of a sequence of 52–55 amino acids in the reductase domain of nNOS and eNOS. The key functions of this insert, determined by the NOS chimeras obtained by its removal, suggest it to be an autoinhibitory element. Moreover, it is AI that largely determines the dependence of NOS activity on Ca^2+^ concentration. For example, AI-deleted eNOS chimeras need a 7-fold lower Ca^2+^ concentration for activation (20 instead of 150 nM) [13].

The AI inhibitory effect is of quite complex nature, which is displayed by its parameters [12, 13]:

1. the overall reducing activity of the NOS reductase domain, which can be estimated from the intensity of the reduction of cytochrome C (as an artificial electron acceptor) bound to FMN;
2. efficiency of electron transfer from NADPH to FAD, estimated from the reduction of ferricyanide bound to FAD;
3. productivity of NO synthesis.

For eNOS and nNOS, CaM binding accelerates the reduction of cytochrome C (total reducing activity of the reductase domain) approximately 10-fold. Initially, iNOS has the same activity (and it is comparable to cytochrome P450), since it is expressed with already bound CaM. In the case of nNOS and eNOS mutants (with AI deleted), the reductase activity without CaM increases radically, 10–30-fold. In the presence of CaM and at physiological concentrations of potassium, sodium, and calcium salts, the reductase activity of the mutant forms is only 3 times higher than that of the original ones [13].

AI has a much weaker effect on the reduction of ferricyanide, i.e. on the transfer of electrons from NADPH to FAD: in the presence of CaM and at physiological salt concentrations, the efficiency of mutant forms is only 2 times higher than the efficiency of normal forms, and, in the absence of CaM, the deletion of AI increases the efficiency 3–6 times. With AI deletion, also a noticeable increase in the maximum NO synthesis occurs, particularly, at physiological concentrations of potassium and sodium salts. Based on the results of experiments with mutant forms of constitutive NOSs with AI deleted, a number of preliminary conclusions can be drawn regarding the principles of its functioning [13]:

1. AI increases the level of Ca^2+^, which is necessary for CaM binding by constitutive NOSs, about 7 times, that is one of the reasons for their significantly greater dependence on Ca^2+^.
2. AI also increases the Ca^2+^ dependence of constitutive NOSs due to the fact that it radically (10– 30-fold) reduces the reductase activity of NOS without bound CaM. In the case of bound CaM, AI reduces this activity much weaker (3-fold).
3. Most likely, there are other factors, beside AI, that determine Ca^2+^ dependence of constitutive NOSs since, despite the enhanced reductase activity of enzymes without AI, they still require CaM binding for NO synthesis. Besides, mutant forms of iNOS with added AI synthesize NO even at Ca^2+^ concentrations well below the threshold.
4. On AI deletion, different NOS isoforms begin to exhibit similar reductase activity, and in CaM-bound mutant forms this activity is higher than that in CaM-associated parent forms.
5. Total autoinhibition of NOS is attenuated by CaM binding both for AI-deleted mutant forms and for normal parent enzymes, and CaM binding enhances the reductase activity of AI-deleted mutant NOS to a greater extent than that of the parent ones.
6. Presumably, the lowest productivity of eNOS compared to other isoforms is due to its most effective AI, which continues to partially inhibit the activity of the enzyme even with bound CaM and sufficient Ca^2+^. In the case with nNOS, the AI inhibitory effect on reductase activity is fully compensated by CaM binding and Ca^2+^ sufficiency. This is confirmed by the fact that on AI deletion, the reductase activity of mutant eNOS equalizes with that of mutant nNOS.

The available data do not yet allow conclusions about the mechanisms of the effect of AI on the reductase activity of NOS. AI can stereometrically prevent cytochrome C and ferricyanide from contacting the reductase domain, and can also induce a conformational change that impedes electron transfer within the reductase domain, similar to the hinge domain in cytochrome P450, which improves electron transfer between flavins [12, 14].

## Regulatory functions of the C-terminal tail

**Figure 3.**
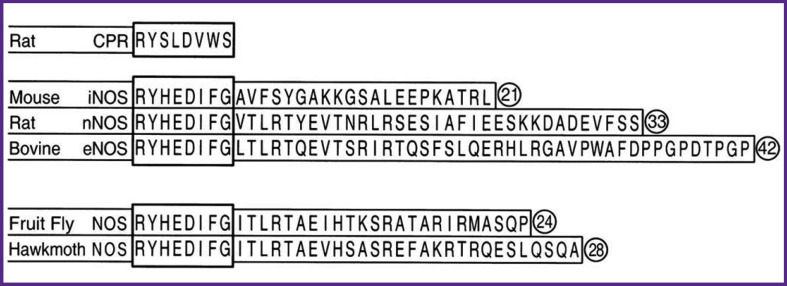
Basic amino acid sequence (tail base, highlighted by a rectangle) and C-terminal amino acid sequences (absent in cytochrome P450 reductase) for mouse iNOS, rat nNOS, bovine eNOS, as well as NOS of fruit fly and hawkmoth [15]

Unlike cytochrome P450 reductase, all NOS isoforms have an extension of 20–40 amino acids at the C-terminus, what is more, the basic sequence of eight amino acids of the C-terminus (base of the tail) have turned out to be absolutely identical for mouse iNOS, rat nNOS, and bovine eNOS, as well as the NOS of the fruit fly and the hawkmoth, and the extensions (the C-tail directly) have different lengths and amino acid sequences [15] (Figure 3).

Experiments with mutant forms of NOS (with the C-tail extended to varying degrees) demonstrated that the C-tail has an inhibitory effect. In contrast, the base of the tail serves for efficient NO synthesis. When both the extension (C-tail) and the base sequence are deleted in iNOS, NO synthesis drops by 70%. When only the extension is deleted, the synthesis of NO increases by 15%, and the reductase activity is enhanced 10-fold. These data were obtained in the presence of CaM, since iNOS could not be expressed without it [12, 15].

Since nNOS and eNOS are easily expressed both with and without bound CaM (by changing the level of Ca^2+^ concentration), the effect of the C-tail can be detected in both cases. For the parent normal enzymes, CaM binding enhances reductase activity 10-fold for nNOS and 3-fold for eNOS. For mutant forms (with the C-tail deleted), CaM binding, on the contrary, slows down the reductase activity by about 36% for nNOS and by 46% for eNOS. At the same time, in the absence of CaM, the reductase activity of mutant eNOS is about 7 times, and nNOS is 21 times greater than the reductase activity of the parent normal constitutive NOS, i.e. CaM works as a partial non-competitive inhibitor of the reductase activity of mutant forms. At the same time, for normal constitutive forms of NOS with a preserved C-tail, CaM acts as a catalyst for reductase activity [15, 16].

As is the case with AI, the regulatory effect of the C-tail on the efficiency of different steps of NO synthesis by constitutive forms of NOS is of a complex nature [15, 16]:

in the presence of CaM, the reductase activity (namely, reduction of cytochrome C bound to FMN, and not electron transfer to heme) for mutant and normal forms of nNOS and eNOS is approximately the same, tail deletion has no radical effect;

in the absence of CaM, with the C-tail deleted, the reductase activity of both isoforms increases 7–21 times, but the rate of NO synthesis for the mutant nNOS is the lowest (perhaps due to the formation of superoxide by dissociation of the heme–Fe(II)–O_2_ complex in the oxidase domain);

the C-tail contributes to the Ca^2+^ dependence of the constitutive forms of NOS, since CaM binding enhances reductase activity in the presence of the C-tail, and in its absence, on the contrary, it diminishes.

It is assumed [15] that the C-tail does not directly interact with the heme domain, AI, and CaM, since these domains are located far from it. Most likely, CaM is located on one side of the flavin wall, while the C-tail covers its other side. At the same time, CaM and the C-tail modulate the electron flow between flavins, coordinating the distance and relative orientation of the isoalloxan rings, and also regulate the electron flow from FMN to the terminal acceptor (cytochrome C, ferricyanide, or heme domain). Experiments with substitution of serine for aspartate in the C-tail showed that CaM binding causes the C-tail shift for a less inhibition of the electron flow, probably, due to repulsive electrostatic forces of negatively charged phosphate or aspartate residues.

## Conclusion

CaM, AI, and the C-tail are the main elements of NOS autoregulation, modulating electron transfer from flavin to heme. CaM binding causes a change in interdomain distances, a shift, and, as a result, a decrease in the inhibitory effect of AI and the C-tail [12, 15, 16]. The effects of CaM, apparently, are realized through the formation of temporary ionic and hydrophobic interactions with the surface of the NOS oxidase domain, which facilitate its interaction with FMN. CaM also shifts the conformational equilibrium of the reductase domain towards more open conformations, reduces the lifetime and the stereometric distribution of conformations, accelerating the transitions between them and the flow of electrons through the reductase domain. AI is likely to induce a conformational change that impedes electron transfer within the reductase domain, similar to the hinge domain in cytochrome P450 [12, 14].

The C-tail together with CaM modulate the electron flow between flavins by changing the distance and relative orientation of isoalloxane rings, and also regulate the electron flow from FMN to the terminal acceptor. The proposed mechanism is the C-tail shift and, as a result, a decrease in its inhibitory effect on electron transfer during CaM binding due to the electrostatic repulsive forces of negatively charged phosphate and aspartate residues.

The functioning of the regulatory elements of NOS requires further study, which will make it possible to detail their complex, multidirectional interaction algorithm. A more complete understanding of the mechanisms of autoregulation of NO synthases in norm and pathology is necessary for modern NOS modeling technologies, which will help identify the pathogenesis of various socially significant diseases, improve the efficiency of their diagnosis and targeted therapy.
